# A Case of a Boy With Undiagnosed Fibrolamellar Carcinoma Who Died Due to Severe Hemorrhage From Diaphragm

**DOI:** 10.7759/cureus.59355

**Published:** 2024-04-30

**Authors:** Haruaki Naito, Yuki Chang, Katsuya Nitta, Eiji Kadota, Yasuhiro Kakiuchi

**Affiliations:** 1 Department of Forensic Medicine, Kindai University, Osakasayama, JPN

**Keywords:** child, legal autopsy, fibrolamellar carcinoma, hemophagocytosis, internal hemorrhage

## Abstract

An 11-year-old boy presented with vomiting and abdominal pain. Ultrasonography and blood tests revealed no abnormalities. He was diagnosed with viral gastroenteritis; however, the following morning, he was found dead in bed. Postmortem examination revealed that a 1,900 mL hemorrhage with strong coagulation from the diaphragm was the cause of death. He had no traumatic episodes, injuries, or a medical history of hemorrhagic diathesis. The presence of a fibrin-like clot indicated coagulation activation; however, most criteria for disseminated intravascular coagulation were not observed. Fibrolamellar carcinoma, a rare hepatocellular carcinoma, was found; however, liver disorder was not estimated based on the pathological findings. In conclusion, the mechanism of hemorrhage could not be explained. Although we were unable to identify the cause of the hemorrhage, we could not completely rule out the possibility that fibrolamellar carcinoma had an unknown influence on the hemorrhage. Given the limited number of studies on fibrolamellar carcinoma, we present a case of a boy with undiagnosed fibrolamellar carcinoma who died due to severe hemorrhage.

## Introduction

Severe organ hemorrhage is sometimes observed during forensic examinations. However, in the absence of traumatic episodes, injuries, or a medical history of hemorrhagic diathesis, identifying the underlying mechanism is challenging. First, in approximately 60% of patients with hemorrhagic diathesis, the mechanism remains unknown despite the examination of coagulation and platelet functions [1,2]. Second, if the hemorrhage is endogenous, the related symptoms and findings should be observed in the lifetime and postmortem periods. However, when the severity and location of the primary hemorrhage are critical, the patient may die quickly, and there may be only mild findings. For example, since disseminated intravascular coagulation (DIC) advances rapidly, and 95% of the fibrin clots melt 6 hours after death, if the primary hemorrhage is critical, finding the typical findings on postmortem examination is challenging [3].

Herein, we report the case of a boy who complained of vomiting and abdominal pain and died the following day. He had no traumatic episodes, injury, or medical history of hemorrhagic diathesis, and an autopsy revealed a 1,900 mL hemorrhage with strong coagulation from the diaphragm.

This case report was previously presented as a poster at the 107th Annual Meeting of Forensic Medicine in Japan on June 8, 2023.

## Case presentation

An 11-year-old boy presented with abdominal and shoulder pain. In the clinic, the patient vomited and felt pain when his upper abdomen was touched. Ultrasonography revealed no significant signs. The pain was resolved by administering intravenous fluid therapy. Blood tests revealed no abnormalities. The patient was diagnosed with viral gastroenteritis. He vomited at night and complained of abdominal pain. The next morning, he was lying face-down and found dead on the bed. He had no traumatic episodes. His medical history indicated that he had *Mycoplasma pneumoniae* when he was seven years old.

**Figure 1:**
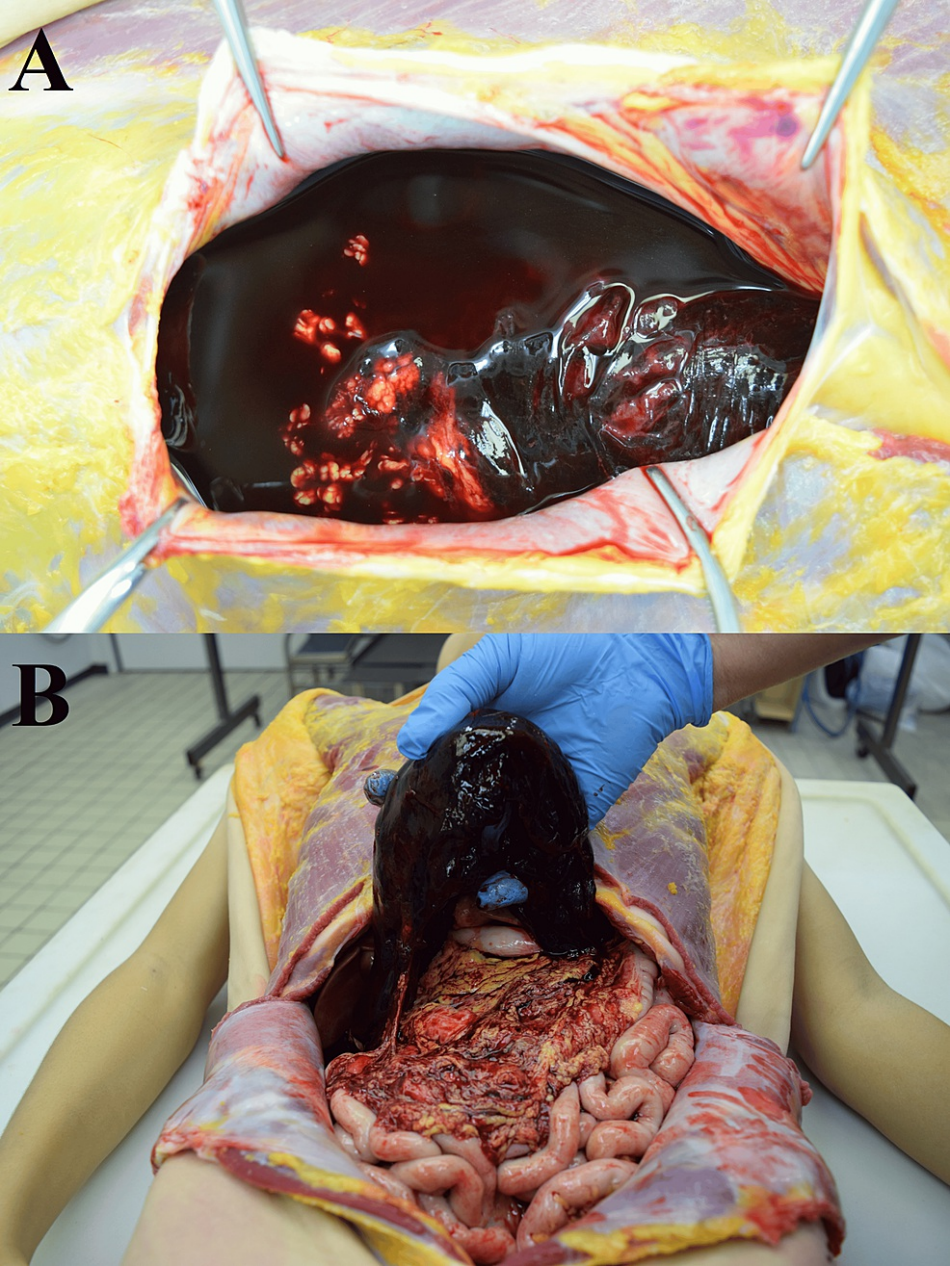
1,900 mL of blood with strong coagulation in the abdominal cavity. (A) there was 1,900 mL of blood in the abdominal cavity. (B) strong coagulation was found in the blood.

**Figure 2:**
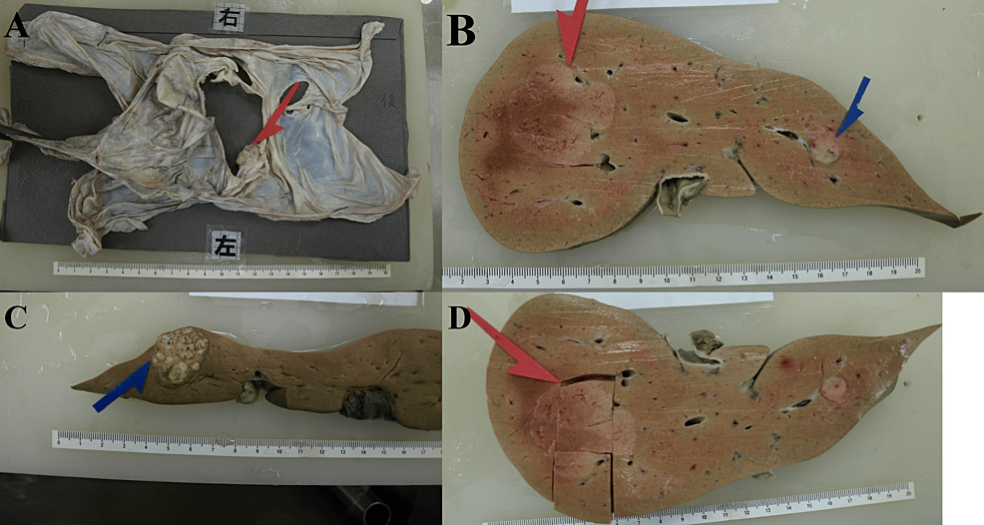
Diaphragm and liver post-formalin fixation. (A) a 4 cm × 4 cm hemorrhage source was observed on the left abdominal side of the diaphragm. (B) a white mass with a diameter of 4.5 cm in the right lobe of the liver, and 1.1 cm and (C) 2.8 cm diameter white masses in the left lobe of the liver were found. (D) The middle of the mass in the right lobe and area above the mass were taken for the histopathological examination.

An autopsy was performed one day after death. The patient’s height and weight were 148 cm and 35 kg, respectively. Moderate rigor mortis was observed in all joints. Livor mortis was reddish and weak on the left side of the face and front thigh, and could not be erased by strong pressure. In the abdominal cavity, 1,900 mL of blood with strong coagulation was observed (Figures 1A-1B). Blood in the heart also showed strong coagulation. All organs were anemic. Splenomegaly was not observed. A 4 cm × 4 cm hemorrhage source was observed on the left abdominal side of the diaphragm (Figure 2A). The diaphragm was the only source of hemorrhage. A white mass with a diameter of 4.5 cm was found in the right lobe of the liver, and 2.8 cm and 1.1 cm diameter white masses were found in the left lobe of the liver (Figures 2B-2C). The middle of the mass in the right lobe and the area above the mass were taken for the histopathological examination (Figure 2D).

The nasal swab was negative for Group A Streptococcus and severe acute respiratory syndrome coronavirus 2 (SARS-CoV-2). Postmortem blood analysis revealed that red blood cells were 123 × 10^4^/μL, hemoglobin was 3.4 g/dL, white blood cells were 110 × 10^2^/μL, and platelet was 22.8 × 10^4^/μL. Thus, pancytopenia was not observed in the case. The C-reactive protein (CRP) levels could not be measured.

**Figure 3:**
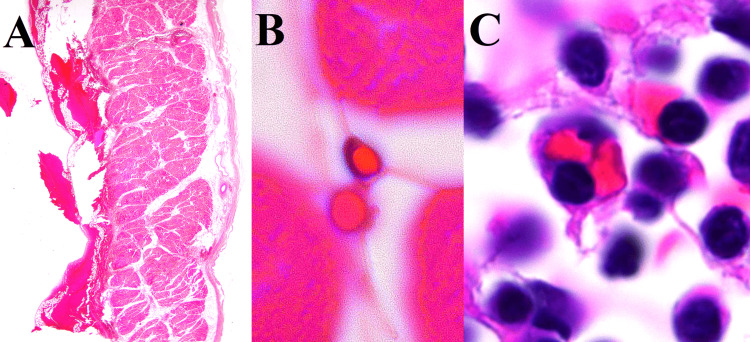
Microscopic morphology of the diaphragm and sternal bone marrow (hematoxylin and eosin). (A) Hemorrhage was found from the serosa on the left side of the abdominal cavity of the diaphragm (10x). (B) Hemophagocytosis was identified in the diaphragmatic hemorrhage and (C) sternal bone marrow by using the immersion lens (1000x).

**Figure 4:**
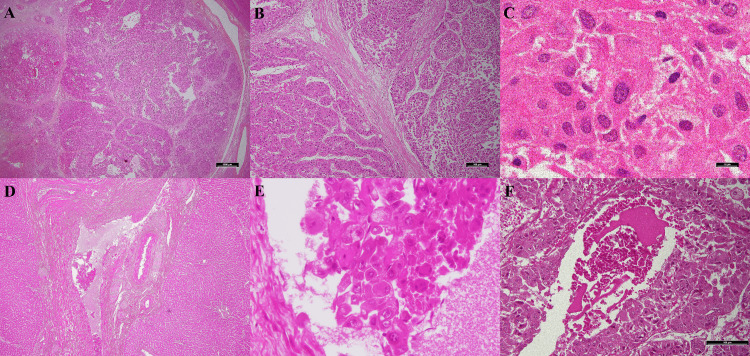
Microscopic morphology of the fibrolamellar carcinoma (hematoxylin and eosin). Lamellated scars between tumor cells are shown at (A) 10x and (B) at 100x magnification. (C) Large polygonal cells and abundant eosinophilic cytoplasms are shown (1000x). (D) at 40x and (E) at 400x magnification, cancer cells invade the bile duct. (F) Necrosis is seen in the fibrolamellar carcinoma (200x).

**Figure 5:**
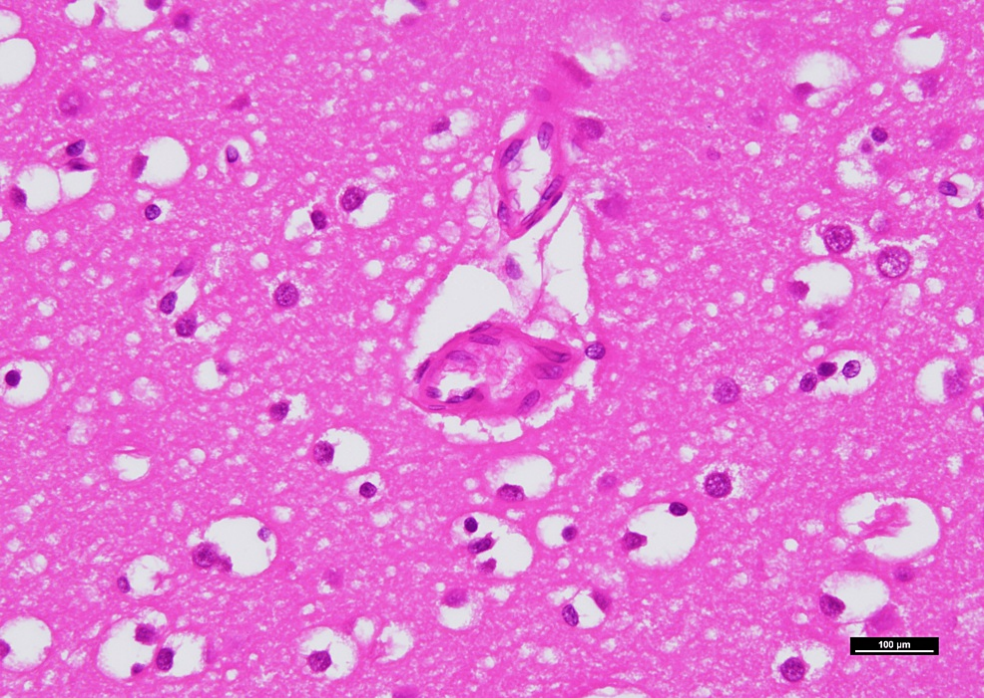
A fibrin-like clot observed in the microvessel of the brain (1000x).

For the histopathological examination, the tissues of the heart, lung, liver, kidney, adrenal gland, spleen, diaphragm, chest lymph nodes, and brain were examined. No histopathological findings associated with the cause of death were found in the heart, lung, liver, kidney, adrenal gland, spleen, and brain tissues. Hemorrhage was observed from the serosa on the left side of the abdominal cavity of the diaphragm. Using the immersion lens, hemophagocytosis was identified in the diaphragmatic hemorrhage and sternal bone marrow (Figures 3A-3C). There were suggestive signs of fibrolamellar carcinoma (FLC) in this liver tissue, such as an eosinophilic nucleus, eosinophilic cytoplasm, island areas of cancer cells divided by central lamellated scars, invasion of the bile ducts, and necrosis (Figures 4A-4F). Necrosis was observed in approximately 3% of the malignant area. A fibrin-like clot was observed in the microvessel of the brain (Figure 5). It was difficult to identify clots in the glomeruli based on postmortem changes. White blood cells, wire loops, and red blood cell casts were not observed in the kidneys. Neutrophils were mainly recruited to the chest lymph nodes.

## Discussion

The cause of death was estimated to be 1,900 mL of hemorrhage. The cause of the hemorrhage appeared to be endogenous as there were no episodes of trauma or external surface findings. We observed a hemorrhage with strong coagulation and a fibrin-like clot in the microvessel of the brain, which indicated coagulopathy concomitant with the hemorrhage [4]. This case report discusses the causes of the hemorrhage, including hemophagocytosis and liver cancer.

First, we discuss the significance of hemophagocytosis in postmortem examinations. Hemophagocytic lymphohistiocytosis (HLH) in children is associated with a high rate of coagulopathy with hemorrhage, such as DIC; however, the sensitivity of hemophagocytosis to hemophagocytic syndrome has been questioned in recent years [5,6]. HLH is a syndrome wherein T cells and macrophages are activated by autoimmune disease or infection, resulting in hyperreactive immune responses [5,7]. If HLH is not treated at an early stage, pancytopenia and vascular endothelial damage can rapidly lead to organ bleeding due to DIC, resulting in death [6,7]. Several studies have stated that hemophagocytosis is not specific to HLH and has no clear relationship to the severity of HLH [6-9]. Except for hemophagocytosis, this case does not meet the criteria of HLH for fever, splenomegaly, pancytopenia, or hepatitis [10]. In this case, hemophagocytosis should be considered a form of mild overreactive immune response.

Because liver failure can cause coagulopathy, we discuss the status of the liver mass. In this case, the liver mass is FLC, which accounts for 1% of primary liver cancers [11-13]. FLC is a rare type of hepatocellular carcinoma primarily identified in individuals aged under 40 and is not associated with preexisting cirrhosis or hepatitis virus [14,15]. It is characterized by large polygonal cells, abundant eosinophilic cytoplasm, lamellated scars between tumor cells, and invasion of the bile duct [13,16]. According to the American Joint Committee on Cancer (AJCC) classification, this patient's FLC is classified as stage IVA. Despite its young age of onset, the prognosis of FLC is currently considered worse than that of conventional hepatocellular carcinoma [11,12,17]. In this case, there were no findings of preexisting liver disease or acute hepatitis. The well-known mechanism of DIC due to accelerated coagulation by tumor necrosis was not suspected, as necrosis is seen in only 3% of tumors [18,19]. Current reports indicate that the symptoms of FLC progress in a manner similar to those of regular HCC [20]. We found no evidence indicating that FLC or liver function was involved in the cause of death.

## Conclusions

We found evidence of an overreactive immune response from hemophagocytosis and neutrophils recruited in the chest lymph nodes. A fibrin-like clot in the microvessel of the brain indicated coagulopathy concomitant with the hemorrhage, but it was hard to estimate the severe inflammation from his symptoms before death. Referring to the conventional hepatocellular carcinoma, the FLC in this case did not seem to impair the liver function or general condition to the point of causing severe inflammation. In conclusion, the mechanism of hemorrhage could not be explained. Although we were unable to identify the cause of the hemorrhage, we could not completely rule out the possibility that FLC had an unknown influence on the hemorrhage. Given the limited number of studies on FLC, we present a case of a boy with undiagnosed FLC who died due to severe hemorrhage.
